# Complete genome sequencing of *Comamonas kerstersii* 8943, a causative agent for peritonitis

**DOI:** 10.1038/sdata.2018.222

**Published:** 2018-11-06

**Authors:** Xiawei Jiang, Wenhong Liu, Beiwen Zheng

**Affiliations:** 1 College of Basic Medical Sciences, Zhejiang Chinese Medical University, Hangzhou, China; 2 State Key Laboratory for Diagnosis and Treatment of Infectious Diseases, Collaborative Innovation Center for Diagnosis and Treatment of Infectious Diseases, The First Affiliated Hospital, College of Medicine, Zhejiang University, Hangzhou, China

**Keywords:** DNA sequencing, Prokaryote, Genome, Bacterial genomics

## Abstract

Because of poor differentiation among the members of genus *Comamonas* using phenotypic methods, human infections caused by *C. kerstersii* are sporadically reported in the literature. Here, we represent the first complete genome sequence of *C. kerstersii* 8943, which caused peritonitis in a patient with continuous ambulatory peritoneal dialysis (CAPD). The complete genome with no gaps was obtained using third-generation Pacific Biosciences (PacBio) RSII sequencing system with single-molecule real-time (SMRT) analysis. Protein-coding genes, rRNAs and tRNAs were predicted. Functional annotations of the genome using different databases revealed several genes related to pathogenicity including antibiotic resistance genes and prophages. Our work demonstrates that whole genome sequencing can enhance the resolution of clinical investigations and our data can be used as a reference genome during the rapid diagnosis of *C. kerstersii* infections in the future.

## Background & Summary

*Comamonas kerstersii*, first described in 2003, has been recognized as non-pathogenic^1^. Human infections caused by *C. kerstersii* were unusual and have been reported only recently. Currently, only five reports of *C. kerstersii*-related infections are available. In 2013, Almuzara *et al.* reported four cases of *C. kerstersii*-induced intra-abdominal infection, which represented the first report of human *C. kerstersii* infections^2^. Shortly after the first report, cases of *C. kerstersii* related bacteraemia and abdominal infections were documented^3,4^. Recently, *C. kerstersii* were reported to be involved in psoas abscess, pelvic peritonitis and acute perforated appendicitis^5,6^. The infections caused by *C. kerstersii* may be underestimated in previous literatures as the phenotypic methods used for the identification of bacteria cannot provide effective measurement for distinguishing between *C. kerstersi* and other *Comamonas* species^2^.

A strain (J29) of *C. kerstersii* was isolated from the dialysis effluent of a patient with continuous ambulatory peritoneal dialysis (CAPD)-peritonitis using sheep blood agar plates. No growth was detected when cultivated anaerobically, indicating that it is a strict aerobe. This strain was initially identified using VITEK 2 system (bioMerieux, France) applying GN ID card. However, the result showed an unidentified organism. Matrix-assisted laser desorption ionization-time of flight mass spectrometry (MALDI-TOF-MS) (Bruker Daltonics, Germany) identified this strain as *C. kerstersii* with a log(score) value of 2.258. To further confirm the bacterial identity, we applied whole genome sequencing, which is a useful tool that enables precise detection of fastidious organisms. Initially, we used Illumina HiSeq platform and ABySS software. However, we only got a draft genome with 148 contigs (ASM129444v1). Because of the importance of and our interest in the study of this strain, we intended to get its complete genome sequence. In the present study, this strain (J29), also termed as 8943, was sequenced using the third-generation Pacific Biosciences (PacBio) RSII sequencing system and a single-molecule real-time (SMRT) analysis. Finally, we obtained the complete genome sequence of this strain. Through RNAmmer, we got the 16S rRNA sequence of *C. kerstersii* 8943 and phylogenetic analysis clearly indicated that this strain shared 100% similarity with *C. kerstersii* LMG 3475^T^ (AJ430347) with regard to 16S rRNA sequence. Our study represented the first complete genome sequence of *C. kerstersii*. Our data reported here will provide the genome reference for diagnosing the presence of *C. kerstersii* in infectious diseases in future, and will be used in the comparative genomic analysis within *Comamonas* genus to elucidate the pathogeny of this bacterium.

## Methods

### DNA extraction

*C. kerstersii* 8943 was cultivated in tryptic soy broth, which contains 17.0 g/L tryptone (pancreatic digest of casein), 3.0 g/L soytone (peptic digest of soybean), 2.5 g/L glucose, 5.0 g/L sodium chloride and 2.5 g/L dipotassium phosphate. After shaking at 37 °C for 24 h, bacterial cells were harvested by centrifugation at 5,000 rpm for 10 min. Genomic DNA was extracted using QIAamp DNA Mini Kit (Qiagen, Germany) according to manufacturer’s instructions. The quality and integrity of genomic DNA was assessed using 1% agarose gel electrophoresis and densitometry compared to the appropriate size standards. Meanwhile, DNA yield and purity were measured using NanoDrop™ 2000 spectrophotometer (Thermo Fisher Scientific, USA) and Qubit®2.0 fluorometer (Thermo Fisher Scientific, USA).

### Whole genome sequencing

Qualified genomic DNA was sheared using a Covaris® g-TUBE® shearing device (Covaris, USA) (>10 kb insert sizes). After shearing, the approximate sizes of the DNA were determined using Agilent® 2100 Bioanalyzer (Agilent Technologies, USA). The fragmented DNA was then purified using 0.45×AMPure® PB beads (Pacific Biosciences, USA). The ends of the fragmented DNA were repaired using the PacBio Template Prep Kit (Pacific Biosciences, USA) before ligating to the hairpins (SMRTbell™ templates). The resulting SMRTbell library was quantitated via Qubit. Before sequencing, sequencing primers were annealed to both ends of the SMRTbell template and DNA sequencing polymerases were bound to the templates to form the template-polymerase complex. Single-molecule real-time (SMRT®) sequencing was performed using a Pacific Biosciences RSII sequencer (PacBio, Menlo Park, CA) according to the manufacturer’s instructuions (MagBead Standard Seq v2 loading, 1×180 min movie) using P4-C2 chemistry.

### Genome assembling and annotation

Hierarchical Genome Assembly Process (HGAP) pipeline was used to a generate high quality *de novo* assembly of the genome with default parameters^7^. Shorter reads were aligned against the longest reads to correct random errors and generate the pre-assembled reads that were both long and of high accuracy. The quality of the assembled genome was assessed using CheckM v1.0.9^8^. The circulation of the assembled genome was verified by aligning the complete genome with the draft genome of *C. kerstersii* 8943 (ASM129444v1). Open reading frames (ORFs) were predicted using Glimmer v3.02. rRNAs and tRNAs were predicted using RNAmmer^9^ and tRNAscan-SE^10^, respectively. The phylogenetic tree was constructed according to the neighbor-joining method using Molecular Evolutionary Genetics Analysis (MEGA) 7.0 software^11^. Antibiotic resistance genes were annotated using BLAST-2.7.1+ program^12^ against the Antibiotic Resistance Gene-ANNOTation (ARG-ANNOT) database^13^ with an e-value cut-off of 1e-5 and identity cut-off of 90%. Prophage regions were identified by PHAge Search Tool Enhanced Release (PHASTER)^14^. The circular map of the genome was generated using DNAPlotter software^15^ and multiple genome alignment was performed using BLAST Ring Image Generator (BRIG)^16^. The genomic average nucleotide identity (ANI) was calculated using Orthologous Average Nucleotide Identity Tool (OAT)^17^.

### Code availability

Most of the custom codes used in the generation or processing of our data are stated in the Methods section. Detailed information including versions of software and database are provided in Table 1.

## Data Records

Whole genome sequence of *C. kerstersii* 8943 has been deposited in GenBank (Data Citation 1). All of the reads for *C. kerstersii* 8943 genome have been deposited in the NCBI Sequence Read Archive (Data Citation 2).

## Technical Validation

**Figure 1:**
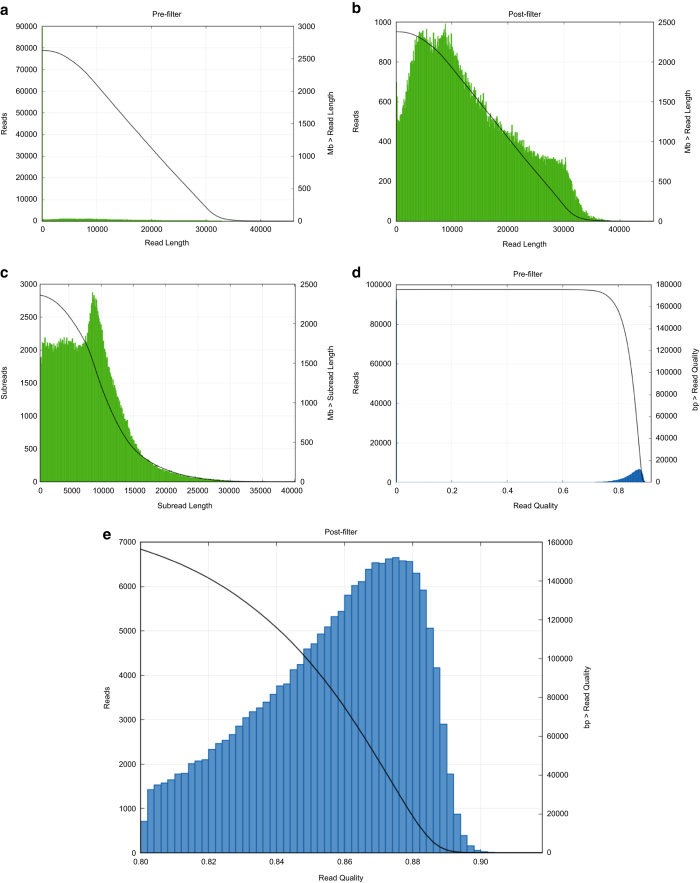
Quality control of the sequencing data. (**a**) Read length distribution before filtering. (**b**) Read length distribution after filtering. (**c**) Subread length distribution after filtering. (**d**) Read quality distribution before filtering. (**e**) Read quality distribution after filtering.

**Figure 2:**
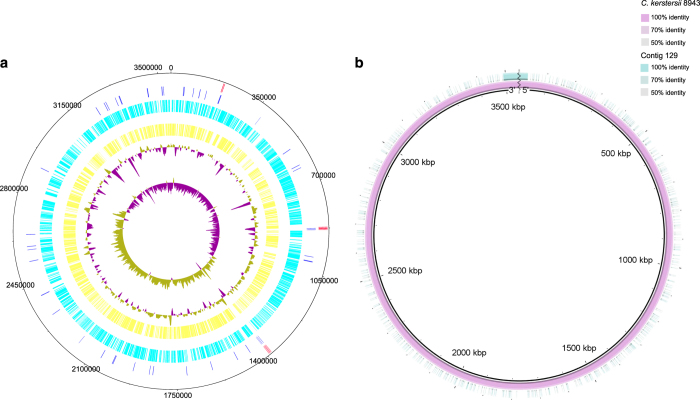
Circular map of *C. kerstersii* 8943 chromosome. (**a**) Genomic features appearing from inside to outsider are as follows: GC skew, GC plot, forward CDS (yellow), reverse CDS (cyan), tRNAs (blue) and rRNAs (red). (**b**) Assembled chromosome of *C. kerstersii* 8943 (pink), contig 129 of ASM129444v1 (green) and divider line of the chromosome (black zigzag line).

**Figure 3:**
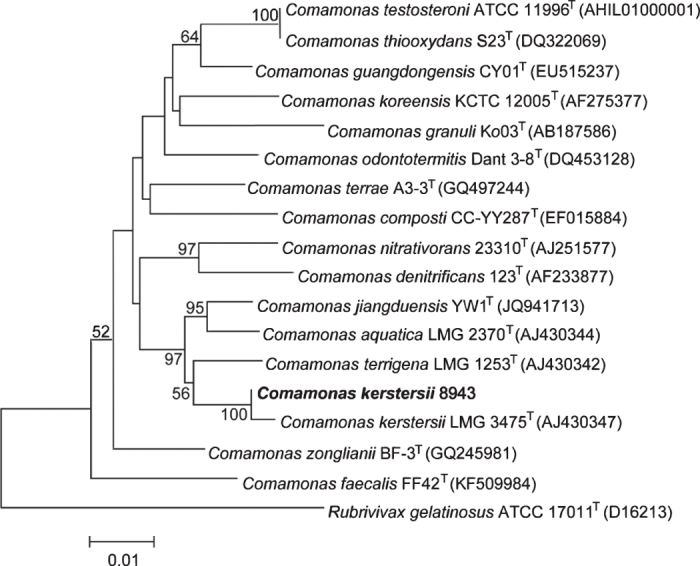
Phylogenetic tree depicting the relationship of *C. kerstersii* 8943 with other *Comamonas* species based on 16S rRNA sequences. The GenBank accession numbers for 16S rRNA of the type strains used in analysis are indicated in parentheses.

To maintain the quality of the assembly, we applied pre-assembly, de novo assembly and assembly polishing steps. Raw reads generated through sequencing were filtered to obtain clean reads, resulting in a total of 300,584 clean reads with an average size of 13,001 bp (Fig. 1). Further, we applied a sub-read filtering step by removing the adapter from the raw reads to obtain clean sub-reads that have a mean length of 7,968 bp and an N50 of 10,227 bp (Fig. 1). The *de novo* assembly generated a chromosome of 3,547,915 bp with a GC content of 59.6%. The genome was predicted to contain 3,155 protein-coding genes, 16 rRNAs and 101 tRNAs (Fig. 2a). The assessment of genome quality showed that the genome exhibited 97.52 % completeness, 0.85 % contamination and 0 % strain heterogeneity, indicating that the assembled genome was of high quality. The alignment of the complete genome with the draft genome of *C. kerstersii* 8943 (ASM129444v1) showed that both the start and the end of the complete genomic sequences were mapping to the same contig (contig 129) of ASM129444v1, indicating that the genome was circular (Fig. 2b). 16S rRNAs were used to construct a phylogenetic tree with other *Comamonas* species and the result reconfirmed the phylogenetic position of this strain as *C. kerstersii* (Fig. 3).

**Figure 4:**
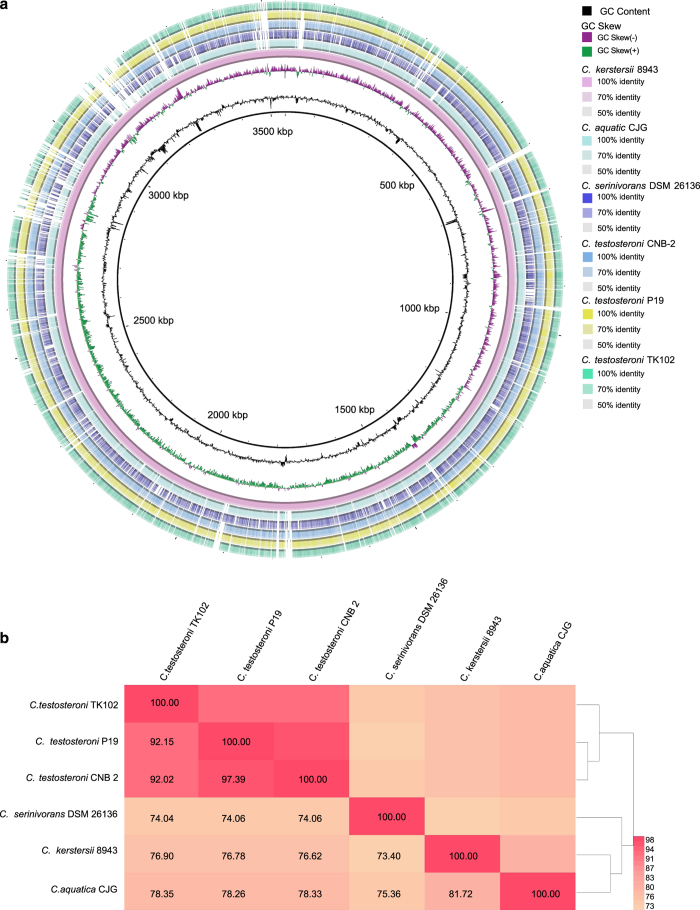
Comparative genomic analysis of *C. kerstersii* 8943 and other *Comamonas* species. (**a**) BLAST atlas of the genomes. (**b**) ANI values between species.

All the protein coding genes could be functionally annotated. A *beta*-lactamase encoding gene, *bla*_OXA-1_, was predicted in the genome. In accordance, *C. kerstersii* 8943 was tested to be resistant to ampicillin, a beta-lactam antibiotic. Besides *C. kerstersii* 8943 reported in the present study, there are five other completed genomes of *Comamonas* species which have been deposited in GenBank. Comparative genomic analysis was conducted with all the six genomes and the results supported the different isolation sources of *C. kerstersii* 8943 (isolated from human) and other *Comamonas* species (isolated from environment) (Fig. 4a). The ANI values among the six genomes indicated that there were great differences between *C. kerstersii* 8943 and other *Comamonas* species (Fig. 4b). The genome size of *C. kerstersii* 8943 was the smallest among the six *Comamonas* species and this was consistent with previous reports, which have shown that symbiotic bacteria usually harbour smaller genomes compared with free-living bacteria^18,19^. As a pathogen, *C. kerstersii* 8943 was found to have more intact prophage regions than other environmentally derived *Comamonas* species (Table 2). Moreover, 10 antibiotic resistance genes with high amino acid identity (>90%) were found in the genome of *C. kerstersii* 8943, whereas no antibiotic resistance genes were annotated in the other five *Comamonas* species (Table 2). These genes, including *tet*A, *str*B, *sul*1, *bla*_OXA-1_, *str*A, *sul*2, *cat*B3 and *flo*R, enable the survival of *C. kerstersii* 8943 in a clinical environment.

In summary, we applied the above-mentioned software and parameters in the quality control of this dataset. Therefore, theresulting data should be error-free. In addition, the annotation analysis performed using this dataset was in accordance with experimental results. Furthermore, comparative genomic studies using this dataset indicate its high level of accuracy and practicability.

## Additional information

**How to cite this article:** Jiang, X. *et al*. Complete genome sequencing of *Comamonas kerstersii* 8943, a causative agent for peritonitis. *Sci. Data*. 5:180222 doi: 10.1038/sdata.2018.222 (2018).

**Publisher’s note:** Springer Nature remains neutral with regard to jurisdictional claims in published maps and institutional affiliations.

## Supplementary Material



## Figures and Tables

**Table 1:** Versions of software and database used in this study.

**Software/Database**	**Application**	**Version/Date**
HGAP	Genome assembly	SMART analysis 2.2.1
CheckM	Genome quality assessment	1.0.9
RNAmmer	rRNA prediction	1.2
tRNAscan-SE	tRNA prediction	1.3
BLAST	Sequence comparison	2.7.1+
ARG-ANNOT	Antibiotic resistance genes prediction	May 2018
PHASTER	Prophages prediction	May 2018
DNAPlotter	Genome map	Artemis 17.0.1
BRIG	Genome alignment	0.95
MEGA	Phylogenetic analysis	7.0.26
OAT	Average nucleotide identity calculation	Jul 2018

**Table 2:** Feature differences between *C. kerstersii* 8943 and other *Comamonas* species.

**Species**	**Isolation source**	**Genome size (bp)**	**Intact prophage regions**	**Antibiotic resistance genes**
*C. kerstersii* 8943	dialysis effluent of a patient	3,547,915	2	10
*C. aquatic* CJG	water	3,764,434	0	0
*C. serinivorans* DSM 26136	compost	4,522,913	1	0
*C. testosteroni* CNB-2	activated sludge	5,373,644	1	0
*C. testosteroni* P19	waste water	5,632,239	1	0
*C. testosteroni* TK102	soil	6,062,703	1	0
